# Digital Health for Equitable Rheumatic Care: Integrating Real-World Experiences to Guide Policy Pathways

**DOI:** 10.3390/healthcare13040438

**Published:** 2025-02-18

**Authors:** Anindita Santosa, James Weiquan Li, Tze Chin Tan

**Affiliations:** 1 Aaria Rheumatology, Gleneagles Medical Centre, 6 Napier Road 08-19, Singapore 258499, Singapore; 2 Department of Gastroenterology and Hepatology, Changi General Hospital, 2 Simei Street 3, Singapore 529889, Singapore; 3 Department of Rheumatology & Immunology, Singapore General Hospital, Singapore 169608, Singapore; tan.tze.chin@singhealth.com.sg

**Keywords:** digital health, rheumatic diseases, global disparities, equity, policy framework

## Abstract

**Background/Objectives**: Digital health technologies show promise in improving rheumatic disease management and reducing healthcare access disparities globally. This paper examines how electronic health records, telemedicine, apps, and wearable devices are used in rheumatic care across different economic settings. **Methods**: The study evaluates digital health technology implementation and outcomes in high-income versus low- and middle-income countries (LMICs). **Results**: Digital health technologies demonstrate improvements in disease monitoring, treatment adherence, and doctor-patient communication, though impact varies by region. Key barriers include poor infrastructure, limited tech literacy, and the digital divide, particularly affecting LMICs. The proposed evidence-based framework recommends strategic investments in digital infrastructure, healthcare system integration, and professional training to address these challenges. **Conclusions**: Implementation of digital health technologies, guided by targeted interventions and regional adaptations, can effectively reduce global disparities in rheumatic care. Success requires sustained commitment to addressing infrastructure and literacy barriers while ensuring equitable access across all economic settings.

## 1. Introduction

Rheumatic diseases, known for their chronic nature and high treatment costs, disproportionately affect the less affluent, thereby inducing a significant health equity gap [1,2]. The health equity gap in rheumatic care in low- to middle-income countries (LMICs) is a multifaceted issue exacerbated by socioeconomic disparities, inadequate healthcare infrastructure, and limited access to specialized care [3]. The disparity in disease outcomes is particularly evident in the management of systemic sclerosis (SSc), idiopathic inflammatory myopathies (IIM), and Takayasu arteritis (TAK), in which high mortality and significant loss of life expectancy are observed [4]. Rural areas face additional challenges, with significant delays in diagnosis due to travel distances and a projected worsening of the rheumatology workforce shortage.

In recent years, digital health interventions have emerged as promising tools to overcome some of these barriers. These technologies, including e-health tools, mobile health (mHealth) applications, and digital patient support programs, have shown potential for improving self-management, enhancing the quality of life, and optimizing clinical outcomes for patients with rheumatic diseases. Studies have demonstrated significant improvements in patient motivation, disease activity, functional ability, and adherence to therapy through digital means [5,6,7]. Remote monitoring using electronic patient-reported outcome measures (ePROMs) has also been effective in managing disease activity and treatment decisions, without increasing clinic visit frequency [8]. Moreover, digital solutions like the Adhera Rheumatology Digital Program have facilitated real-time monitoring and remote management of disease flares and medication issues, with a significant portion of alerts being managed remotely [9]. Patient education through digital platforms has been well received, offering flexibility and the ability to learn in familiar surroundings, although some patients miss the relational support from healthcare providers. Additionally, the development of apps like RheumaBuddy 4.0, which incorporates evidence-based self-management interventions, further supports individualized patient care [10,11,12]. Integrating Internet of Medical Things (IoMT) and multi-modal signal monitoring can further enhance the accuracy and timeliness of diagnoses, reducing human errors and improving patient outcomes [13].

Despite the promise of these digital interventions, their successful implementation faces significant challenges. Issues such as the digital divide, privacy concerns, and the variability in technology access and utilization across different demographic groups could potentially widen the existing health disparities [14]. The pandemic disrupted healthcare services and medication supplies, worsening disease outcomes, particularly among socially vulnerable groups. In Africa, the disparity is stark, with a severe shortage of rheumatologists and healthcare personnel, compounded by political instability, poverty, and endemic diseases, leading to a significant burden of musculoskeletal disorders [15,16]. To mitigate these risks, it is crucial to develop multi-level, context-specific interventions, engage in intersectoral partnerships, and empower historically marginalized groups in the development and implementation of digital health solutions.

This perspective paper examines how digital health technologies can enhance equity in rheumatic care. Drawing from pilot studies in diverse clinical settings, we analyze digital interventions that demonstrate promise in bridging healthcare disparities. Through synthesis of implementation experiences and outcomes, we identify effective strategies and implementation challenges. The recommendations emphasize critical policy enablers—including infrastructure development, regulatory frameworks, and community engagement approaches—essential for sustainable scaling of digital health solutions in resource-constrained environments.

## 2. Methods

This study employs a comprehensive literature review methodology to evaluate the integration of digital health technologies in rheumatic care. A systematic search was conducted across multiple databases, including PubMed, Scopus, and Google Scholar, utilizing keywords such as “digital health”, “rheumatic diseases”, “telemedicine”, “mobile health applications”, and “wearable devices”. Peer-reviewed articles published in the last 10 years were selected based on inclusion criteria focused on the application of these technologies in both high-income and low- and middle-income countries (LMICs). Relevant information was extracted using a structured data extraction form, capturing details such as author(s), year of publication, type of digital health intervention, study population, outcomes measured, and contextual factors. The quality of the included studies was assessed using established criteria to ensure reliability and validity. The synthesized findings provided an overview of the current state of digital health interventions, highlighting benefits and limitations, particularly concerning disparities in access and effectiveness. Based on these insights, this study outlines potential policy implications and recommendations aimed at enhancing the integration of digital health technologies in rheumatic care, including strategies for improving digital infrastructure, increasing digital literacy, and promoting equitable access to technology in LMICs.

## 3. Results

### 3.1. Inequities in Rheumatic Care

Inequities in rheumatic care are pervasive and complex, manifesting across different aspects of healthcare, including access, treatment, and outcomes among diverse demographic groups. During the COVID-19 pandemic, telemedicine emerged as a critical tool in healthcare delivery, but its benefits were not uniformly realized. Racial and ethnic minorities, along with individuals from lower socioeconomic backgrounds, have been less likely to utilize these services, thus widening existing disparities in rheumatology care [17,18,19]. The disparities extend to pediatric rheumatic diseases, which exhibit varying prevalence and outcomes among populations of non-European descent. These differences are not merely statistical but are deeply rooted in a confluence of social and biological factors, including historical trauma, which collectively influence health outcomes [18].

The digital divide in LMICs presents a multifaceted challenge with significant regional variations. In sub-Saharan Africa, political instability and frequent internet shutdowns, especially in countries like Sudan and Ethiopia, severely disrupt the development of digital infrastructure and healthcare delivery [20,21]. Healthcare systems in Southeast Asia encounter unique challenges due to fragmentation and notable rural–urban disparities in digital literacy and access to infrastructure [21]. Cultural factors introduce additional complexity, as evidenced by gender-based disparities in digital access within South Asian communities that restrict women’s participation in digital health initiatives. The interaction of these regional factors demands customized approaches instead of one-size-fits-all solutions [22].

Minority patients frequently encounter multiple barriers at individual, provider, and system levels, resulting in poorer health outcomes and delayed access to treatments [23]. A similar pattern is observed in osteoarthritis management, particularly concerning the underutilization of total joint replacement surgeries among vulnerable populations, influenced by factors such as socioeconomic status, gender, and physician biases [14]. The situation is exacerbated in early inflammatory arthritis, where Black, Asian, and Minority Ethnic (BAME) patients report worse outcomes compared to their White counterparts, despite receiving similar levels of care. This discrepancy highlights the urgent need for healthcare systems to redesign care pathways to better address and mitigate these inequities [24].

**Figure 1:**
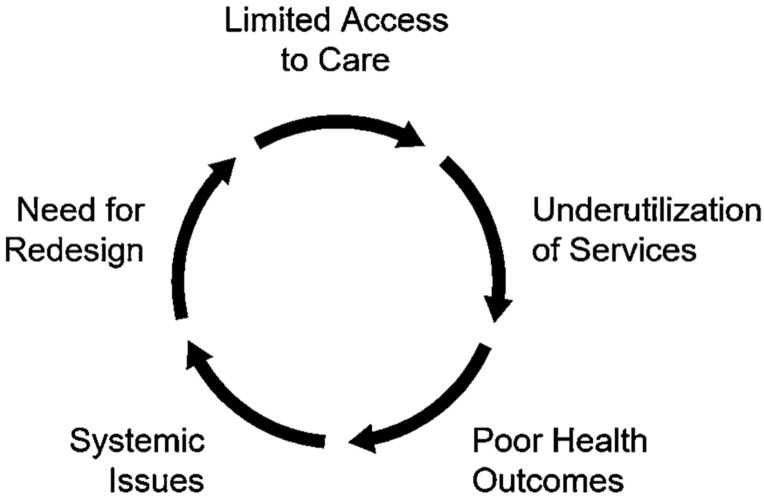
Cycle of Inequities in Rheumatic Care.

Broader systemic issues also play a role. The chronic nature of rheumatic diseases and the absence of distributive justice within healthcare systems complicate the pursuit of equitable care, necessitating a consistent, long-term approach to management [24,25,26]. Additionally, socioeconomic deprivation has been linked to poorer responses to TNF inhibitor treatments, with the most deprived patients exhibiting higher disease activity and more frequent discontinuation of treatment, primarily due to ineffectiveness rather than adverse events [27]. Figure 1 illustrates the cyclical relationship between healthcare system challenges that perpetuate disparities in care delivery. The cycle begins with limited access to care, which leads to underutilization of available services. This underutilization subsequently results in poor health outcomes among affected populations. These poor outcomes contribute to broader systemic issues within healthcare delivery, which in turn create a need for system redesign. Without intervention, this cycle continues as the need for redesign circles back to reinforce limited access to care.

### 3.2. Sphere of Digital Health Interventions in Rheumatic Care

**Figure 2:**
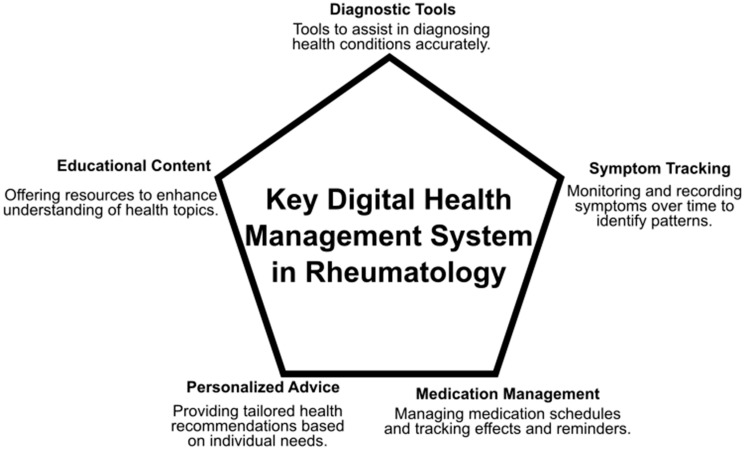
Key Digital Health Management System Components in Rheumatology.

Table 1 and Figure 2 illustrate the principal components of a digital health management system for rheumatology, encompassing diagnostic tools, educational resources, symptom monitoring, medication management, and individualized recommendations. The subsequent discussion elucidates key aspects of digital healthcare in detail.

The scalability and sustainability of DHTs in LMICs face numerous implementation challenges. Infrastructure constraints, particularly unreliable electricity and internet connectivity in rural healthcare facilities, significantly hinder DHT deployment [28]. Financial sustainability remains a crucial obstacle, with many initiatives failing after the pilot phase due to ambiguous funding models [29]. Workforce capacity poses another major barrier, as many facilities lack trained personnel to support digital systems. Evidence from Ghana shows that successful scaling necessitates integrated approaches that simultaneously address both technical and human resource capabilities.

Figure 2 illustrates a Digital Health Management System for rheumatology care, depicted as a pentagon with five interconnected components. At its core, diagnostic tools enable precise identification of rheumatological conditions, paired with symptom tracking for continuous monitoring, flare detection, and treatment assessment. The system includes a medication management module to improve adherence through scheduling, reminders, and outcome monitoring. A personalized advice feature delivers tailored health recommendations, while an educational component provides evidence-based resources to empower patients with knowledge. Together, these components form an integrated solution, fostering collaboration between providers and patients and combining clinical tools with education to optimize rheumatology care.

#### 3.2.1. Telemedicine and Remote Consultations

Telemedicine has significantly influenced rheumatology by maintaining continuity of care and minimizing the need for in-person consultations. In HICs, telemedicine has been seamlessly integrated into healthcare systems, facilitating patient monitoring, triage, and educational efforts. Patients generally exhibit favorable attitudes towards remote consultations, especially for follow-up visits, which are perceived as less burdensome compared to traditional visits [30,31]. However, challenges such as building trust, ensuring accurate assessments, and managing the nuances of chronic rheumatic diseases persist. Younger patients and those with shorter disease durations tend to show better adherence and outcomes in these settings [30]. In LMICs, telemedicine holds significant potential to bridge healthcare access gaps, particularly in regions with low medical density and in underserved areas. The French TeleRheumatology project exemplifies coordinated efforts to improve access to rheumatologic care via dedicated telehealth platforms. However, disparities in access due to socioeconomic and technological barriers pose significant challenges [14,32]. Concerns about the accuracy of diagnostics and the need for physical examinations, especially in complex cases like systemic lupus erythematosus (SLE), highlight the limitations of telemedicine in these settings.

While telemedicine is appreciated for its convenience and ability to reduce routine consultations, ensuring comprehensive care requires a balance with traditional face-to-face visits. This balance is crucial for maintaining the quality of patient–provider relationships and for performing comprehensive assessments that remote consultations cannot achieve [33]. Both HICs and LMICs face the need for enhanced training in telemedicine. Educational initiatives designed to increase healthcare providers’ proficiency in delivering virtual care are essential for integrating telemedicine into medical education and clinical practice effectively [34].

#### 3.2.2. Artificial Intelligence and Machine Learning

Artificial intelligence (AI) and machine learning (ML) are particularly influential in both high-income countries and LMICs, though their application and impact vary significantly due to differences in technological infrastructure, healthcare resources, and data availability. In HICs, AI and ML are integrated into healthcare systems to enhance clinical decision-making and patient management. Advanced algorithms, including deep learning, analyze unstructured data like images and text to detect joint erosions and predict disease activity, greatly improving diagnostic accuracy and treatment approaches [35,36]. AI models, such as the Arithmetic Optimization Algorithm with Deep Learning (ARAC-AOADL), offer high precision in classifying diseases like rheumatoid arthritis (RA), demonstrating the potential for timely and accurate diagnoses. However, challenges persist, including the “black box” nature of AI algorithms, potential biases in data, and the need for extensive, well-labeled datasets for training and validation [37]. In LMICs, AI has the potential to significantly bridge healthcare gaps by providing reliable diagnostic tools that require minimal human intervention. This is crucial in regions with scarce healthcare resources and low medical density. AI and ML can improve the efficiency of diagnoses and managing diseases like RA through technologies such as support vector machines and random forests, which help compensate for the lack of specialized healthcare professionals [38].

Across both HICs and LMICs, AI and ML face several challenges, including the need for external validation to ensure the reliability of the technologies and the involvement of primary stakeholders in model development to ensure clinical relevance and applicability. The increasing availability of biomedical data and the advancement of analytical techniques enhance the potential for precision medicine in rheumatology, particularly in LMICs where access to specialized care may be limited [35,39].

#### 3.2.3. Mobile Health Applications and Wearables LMIC

Mobile health (mHealth) applications support not only continuous monitoring but also proactive management of rheumatic diseases, making healthcare more accessible and personalized. In HICs, mHealth applications like “Rheumatology Connect” and SCQM’s iDialog and COmPASS have been effectively integrated into clinical workflows. These platforms provide educational resources, facilitate disease tracking, and enable direct communication between patients and healthcare providers, significantly improving patient engagement and satisfaction [40,41]. Furthermore, wearable devices employed in these regions for monitoring vital signs and physical activity have shown high user satisfaction and feasibility, although challenges in integration with electronic medical records persist [42].

In LMICs, the growth of mHealth is particularly notable. Digital health applications have proven effective in monitoring patient-reported outcomes, which can significantly improve disease control and patient management in areas where healthcare resources are sparse [43]. However, the adoption faces challenges such as limited access to technology, high costs, and the need for designs that are user-friendly and adapted to local contexts [44]. Ensuring the involvement of patients and clinicians in the design process is crucial for enhancing the usability and effectiveness of these technologies [45]. mHealth applications and wearables provide a vital service in LMICs by delivering remote consultations and continuous health monitoring. This technology reduces the need for physical visits, which is particularly beneficial in remote or underserved areas. Nonetheless, significant issues such as data privacy, affordability, and the digital divide need addressing to ensure these technologies benefit all population segments. The potential of mHealth and wearables to revolutionize clinical research and patient care is immense. Their ability to collect real-time biometric data offers new disease prevention, diagnosis, and management avenues.

### 3.3. Patient Experiences and Insights on Digital Health Interventions in Rheumatic Care

Digital health interventions significantly enhance patient experiences in managing rheumatic diseases by providing targeted, interactive tools. Apps like Arthritis Tracker and My Arthritis offer features for symptom tracking and personalized advice, empowering patients to understand better and manage their conditions. RA Monitor enhances communication between patients and specialists, enabling efficient symptom and treatment tracking. RheumaHelper provides comprehensive tools for both healthcare providers and patients, facilitating improved clinical decision-making. Overall, these apps illustrate the critical role of digital tools in improving healthcare outcomes and patient empowerment in rheumatology, highlighting a shift towards more patient-centered care approaches. However, the reception and effectiveness of these technologies differ significantly, influenced by local healthcare infrastructure, patient needs, and technology availability. In HICs, patients have generally responded positively to digital health tools, appreciating their flexibility, ease of use, and enhanced ability to manage conditions from home. For example, the WebRA trial highlighted the benefits of e-learning programs in facilitating self-management, particularly valuable immediately after diagnosis. Despite the advantages, some patients reported missing the relational support typically provided by face-to-face interactions with healthcare providers [46]. Similarly, tools like the ‘Rheumatic?’ symptom checker were praised for their utility, though some concerns were raised regarding the extensiveness of the questions asked [47]. The TELERA study further underscored the utility of digital tools such as apps for electronic patient-reported outcomes (ePROs) and self-administered tests, noting their ease of use and helpfulness in disease self-assessment while also acknowledging the limitations posed by reduced personal contact [48].

Analysis of patient outcomes reveals significant disparities between HICs and LMICs in the implementation of digital health. Technical and infrastructure barriers in LMICs, such as inadequate internet connectivity and limited access to digital devices, greatly impact the effectiveness of telemedicine. Socioeconomic disparities and lower eHealth literacy in LMICs further influence the adoption and utilization of digital health tools. Regulatory challenges, including the absence of standardized telemedicine policies, hinder widespread adoption. Nevertheless, innovative solutions like assisted telemedicine models demonstrate promise in improving access and outcomes in resource limited.

The COVID-19 pandemic significantly accelerated the adoption of telemedicine, revealing both its benefits and limitations. While telemedicine was found convenient and helped maintain continuity of care, concerns about diagnostic accuracy and the quality of patient–clinician relationships were prominent [49]. In LMICs, digital health interventions like those delivered by Wellthy Care have demonstrated significant improvements in emotional wellbeing and quality of life for patients with RA, showcasing the potential of digital solutions to bridge care delivery gaps [50]. These programs, however, depend heavily on patient adherence and the seamless integration of digital tools into everyday healthcare practices [51].

Across both settings, while digital health interventions offer promising benefits, they must be carefully tailored to meet the diverse needs of patients. This includes ensuring technological accessibility and maintaining essential human interactions, which are critical for patient satisfaction and the effectiveness of care [52,53]. Challenges remain in integrating these tools within existing healthcare frameworks and ensuring that they address both patient concerns and provider capabilities adequately.

### 3.4. Identification of Barriers and Challenges

The identification of barriers and challenges in managing rheumatic diseases reveals significant disparities between LMICs and HICs (Table 2, Table 3 and Table 4). Table 2 elucidates the key differences in the adoption and application of digital health technologies. In HICs, advanced technologies such as electronic health records (EHRs), telemedicine, and wearable devices are more prevalent, enhancing data quality, patient management, and remote monitoring. Conversely, LMICs predominantly utilize mobile health applications, basic telemedicine, and limited EHR capabilities, focusing on expanding access to healthcare services and symptom tracking. Both settings encounter challenges, including inadequate integration, poor user engagement, and technological disparities in HICs, as well as high costs, infrastructure deficits, and digital literacy barriers in LMICs. Opportunities for improvement encompass enhancing interoperability, implementing patient engagement strategies, and addressing the digital divide through targeted policies and funding in LMICs.

Table 3 provides a comprehensive analysis of the current utilization and challenges associated with digital health implementation. High-income countries have extensively integrated digital tools for real-time monitoring and remote consultations, resulting in effective management of disease activity and a reduction in in-person visits. In LMICs, the adoption of digital tools is emerging, albeit frequently impeded by infrastructural limitations, leading to less frequent and effective utilization of real-time data for disease management. Primary challenges in HICs include ensuring seamless integration and sustained user engagement, while LMICs face significant constraints such as shortages of rheumatologists, limited healthcare resources, and lower levels of digital literacy.

Table 4 offers a global perspective on the diverse array of digital health tools employed in rheumatology, detailing their specific functions and target demographics. These technological solutions span from mobile applications designed for symptom tracking and medication management to online communities facilitating patient support and educational platforms tailored for healthcare practitioners. The development and implementation of these digital health innovations are predominantly concentrated in nations such as the United States, United Kingdom, Canada, Germany, Denmark, and Singapore, reflecting the varying degrees of digital health integration and advancement across different countries in the field of rheumatology.

The above comparative analysis elucidates the substantial disparities in the adoption and utilization of digital health technologies in rheumatology between HICs and LMICs. While HICs have demonstrated significant progress in integrating advanced digital solutions, LMICs encounter challenges in adapting and scaling these technologies to their local contexts. Addressing the digital divide, enhancing infrastructure, and fostering collaborative efforts are essential to ensure equitable access and effective digital health implementation in rheumatology care globally. Each setting presents distinct obstacles that influence the efficacy of disease management strategies, necessitating tailored approaches to overcome these challenges.

In LMICs, several critical barriers hinder effective rheumatic care. Access to healthcare resources is often limited, with high out-of-pocket costs for medications and a poor availability of diagnostic tools and treatments significantly affecting patient care, especially for conditions like rheumatic heart disease (RHD). For example, in Uganda, the challenges extend into pediatric rheumatology, where resources are even scarcer [54]. The implementation of effective screening programs, such as those for RHD, is often hampered by the lack of human and financial resources, despite the availability of advanced echocardiographic screening technologies [55]. Moreover, psychosocial barriers like stigmatization and low self-esteem complicate disease management further, impacting patient outcomes [56]. Conversely, HICs encounter different sets of challenges. Issues such as medication risk aversion and financial burdens associated with care, particularly for RA, frequently hinder achieving optimal disease control [57,58]. Additionally, suboptimal patient–physician communication can impede effective treatment strategies. Unique challenges also arise in the care of transgender and gender diverse individuals with rheumatic diseases, who may face psychosocial barriers and require more empathetic approaches from healthcare providers [59,60].

Both LMICs and HICs share common challenges, including the unpredictability of disease progression and the overarching need for enhanced patient education and support systems. These issues necessitate comprehensive strategies to improve disease management across all socioeconomic settings [61,62]. The COVID-19 pandemic has further exacerbated these challenges, increased the vulnerability of immunocompromised patients and necessitating stringent self-isolation measures. This situation has highlighted the critical need for resilient healthcare systems that can maintain continuity of care under such pressures. Addressing these multifaceted barriers requires a comprehensive approach that includes improving healthcare infrastructure, enhancing access to medications, bolstering patient education, and developing supportive policies. These measures should aim to mitigate financial burdens and address psychosocial impacts, thereby improving the overall management of rheumatic diseases.

### 3.5. Identifying Key Stakeholders and Their Roles

Identifying key stakeholders and defining their roles in managing rheumatic and musculoskeletal diseases (RMDs) requires a nuanced understanding of the various actors across different healthcare settings in both LMICs and HICs. In LMICs, stakeholders such as government health departments, local healthcare providers, community health workers, and non-governmental organizations (NGOs) play critical roles in policy-making, service delivery, and patient education. These stakeholders are pivotal in implementing and sustaining RMD management programs under resource constraints [63]. Conversely, in HICs, the stakeholder landscape includes specialized roles like advanced nursing practitioners and interdisciplinary teams that contribute significantly to patient care outcomes [64].

Both LMICs and HICs benefit from the active involvement of patients and their families, who provide essential insights into the lived experiences of those affected by RMDs, influencing care strategies and outcomes [65]. Effective stakeholder engagement in these settings requires systematic identification and categorization through methodologies such as snowball sampling and iterative processes to ensure inclusion of diverse perspectives from the outset [66,67]. Moreover, stakeholders play crucial roles in research and dissemination, helping to translate evidence into practice and ensuring effective communication of findings to all relevant parties [68]. The integration of these stakeholders into a cohesive framework significantly enhances the effectiveness of RMD interventions, ensuring that both immediate and long-term needs of patients are met across various socioeconomic contexts [69].

Additionally, digital health apps have significant potential to support self-management in regions like sub-Saharan Africa. However, the lack of regulatory standards and guidance poses a major barrier. For these apps to be safely and effectively integrated into healthcare, increased regulatory attention is necessary [70]. In the digital health realm, key stakeholders include healthcare professionals, patients, policymakers, technology providers, and scientific societies. Their involvement is crucial for crafting supportive frameworks and guidelines that promote the adoption of digital health technologies. Furthermore, addressing the unique challenges of diseases like RHD in LMICs requires sustainable, scalable interventions, supported by effective stakeholder engagement to ensure comprehensive planning and execution of digital health strategies, ultimately improving health outcomes and efficiency in healthcare systems.

Table 5 elucidates the prevalent challenges encountered by both LMICs and high-income countries in rheumatology care, as well as the technological advancements and regulatory requirements necessary to address these issues. While LMICs contend with inadequate healthcare infrastructure and a paucity of specialists, HICs face the challenge of implementing existing guidelines effectively. Both settings can potentially benefit from advancements in EHR, AI, and biobanking to enhance diagnosis and treatment. Regulatory frameworks are requisite to ensure the safe and efficacious utilization of these technologies, particularly in domains such as health applications, wearable devices, and AI-driven patient engagement strategies, as well as to address concerns regarding opioid management and algorithmic bias.

## 4. Discussion

### 4.1. Improving the Infrastructure and Technology for Bridging the Digital Divide at the Global Level

Improving digital health infrastructure in rheumatic care is essential for bridging the digital divide and enhancing equitable access to medical services, both in HICs and LMICs. The integration of digital health technologies (DHTs) such as telemedicine, mobile health applications, and wearable devices has shown potential to revolutionize care delivery, yet significant disparities remain in their adoption and impact across different regions.

In HICs, advancements like the Rheumatology Informatics System for Effectiveness (RISE) registry demonstrate how EHRs can be leveraged to improve data collection, quality measurement, and overall care quality [41,71,72]. However, challenges persist in fully transforming rheumatology care delivery due to issues such as inadequate integration of digital tools and poor user engagement [73]. Despite these challenges, programs like the semi-automated telemedicine referral system have successfully reduced wait times and enhanced efficiency in HICs, underlining the importance of thoughtful implementation and IT infrastructure optimization [74]. Conversely, in LMICs, the need to address the digital divide is even more critical due to disparities in access among racial/ethnic minority groups and those with lower socioeconomic status, which have been further exacerbated by the COVID-19 pandemic. Essential initiatives include enhancing robust infrastructure and ensuring the widespread availability of technology in underserved areas. Tools such as the REMORA app, which connects daily symptom tracking to EHRs, offer promising avenues for improving clinical decision-making and disease management but require adaptations to meet the diverse needs of patients, including those with physical disabilities or language barriers [75,76].

Furthermore, the integration of DHT such as electronic patient-reported outcomes (ePROs) and voice-enabled systems can significantly improve patient engagement and monitoring, particularly beneficial for those suffering from conditions like hand arthritis. Platforms like Adhera for Rheumatology illustrate the feasibility of real-time monitoring of disease outcomes, although maintaining long-term patient engagement continues to be a challenge [77]. To truly bridge the digital divide in rheumatic care, it is crucial to overcome technological and systemic barriers, including the development of personalized data-driven approaches powered by AI and big data. Such technologies can empower clinicians and patients alike but require a concerted effort to ensure their effective integration into healthcare systems.

### 4.2. Regulatory Frameworks to Avoid Misuse and Catalyze Faster Dissemination

Regulatory frameworks for managing RMDs vary significantly between LMICs and HICs, influencing the effectiveness of disease management. In LMICs, the challenges are compounded by inadequate healthcare infrastructure and a scarcity of rheumatology specialists, which delay diagnosis and treatment, leading to worse health outcomes [78,79]. Both LMICs and HICs face issues in implementing clinical guidelines and integrating lifestyle management into care strategies crucial for slowing disease progression [80,81]. Additionally, the management of opioid dependence within RMD care varies, with differing definitions and standards for long-term opioid use across regions, further complicating treatment approaches.

The integration of digital health technologies has made significant advancements through machine learning applications in rheumatology. Convolutional neural networks have shown high diagnostic accuracy, achieving macro AUCs of 92% for erosions and 85% for synovitis in hand MRI analysis of inflammatory arthritis [82]. For synovitis scoring in ultrasound images, CNNs have reached a four-class accuracy of 77.6% [83,84]. Machine learning models that utilize electronic health records and multi-omics data have demonstrated promising results, with AUCs exceeding 0.85 in identifying RA patients [85]. However, implementation challenges persist. Algorithmic bias stemming from limited training data diversity impacts model generalizability (Shi et al., 2024 [85]), while interconnected health systems create data security concerns [86]. Real-world implementations have shown success: the AI-assisted system at Leeds Teaching Hospitals reduced reading time by 40%, and the integration of machine learning in the Swedish Rheumatology Quality Registry has improved treatment decisions. While these technologies appear promising, clinician expertise remains essential, as AI tools complement rather than replace clinical judgment [87].

Technological advancements such as EHRs, biobanking, and AI offer significant potential to enhance RMD diagnosis and treatment. However, their implementation is more feasible in HICs, which possess the necessary resources [88]. Collaborative efforts led by research institutions and patient organizations are vital in bridging these disparities, advocating for patient needs, and facilitating the exchange of best practices globally [89]. The role of rheumatology nurses is also crucial in providing patient-centered care and managing therapies to ensure continuity of care.

### 4.3. Standards and Guidelines for Digital Health in Rheumatic Care

The integration of DHT in RMD care presents a promising avenue to enhance management and patient outcomes globally, though challenges persist across varying regions. In HICs, digital tools like electronic patient-reported outcomes (ePROs) and telehealth have been more readily integrated, offering real-time monitoring and remote consultation capabilities that alleviate healthcare system burdens and reduce the need for in-person visits. These technologies have shown efficacy in monitoring disease activity and managing flares, thus enhancing patient care [9,80].

Conversely, LMICs face significant barriers due to shortages of specialized healthcare providers, limited healthcare infrastructure, and lower digital literacy levels, which impede the widespread adoption and effective utilization of digital health solutions [9,81,90]. Despite these challenges, initiatives like those led by the European Alliance of Associations for Rheumatology (EULAR) are striving to bridge these gaps through international collaboration, providing necessary training, resources, and support to facilitate digital health implementation in LMICs [89,91]. The COVID-19 pandemic has accelerated the shift towards remote care models, underscoring the urgent need for standardized guidelines to ensure the effective and equitable use of DHT in rheumatic care across different regions. Implementation science provides a valuable framework to enhance the integration of these digital tools into routine clinical practice by identifying and addressing barriers, and leveraging facilitators to optimize care quality for patients with RMDs globally.

To harness the full potential of digital health in rheumatology, it is imperative to develop and enforce global standards and guidelines that accommodate the unique challenges and needs of both LMICs and HICs. This approach should focus on ensuring accessibility, enhancing user engagement, and maintaining data security, ultimately facilitating better disease management and improving health outcomes for all patients, irrespective of geographic or economic constraints.

### 4.4. Policies to Support Digital Health Adoption

Most digital tools are developed in high-income countries, tested, and validated in settings and languages biased towards HIC. For their full potential to be realized, especially in LMICs, there is a need for higher quality, user-centered applications that cater to the specific needs and conditions of local populations. Table 1 illustrates a range of mobile apps, social networks, patient education websites, and chatbots designed to support patients with various rheumatic diseases and healthcare professionals. However, the effectiveness of these tools in bridging the digital divide is contingent upon several factors, including accessibility to technology, geographic distribution of resources, language barriers, digital literacy skills, and healthcare professional adoption. To effectively address the digital divide, concerted efforts must be made to improve access to technology, provide multilingual resources, offer digital literacy training, and ensure equitable distribution of resources across diverse geographic regions and socioeconomic groups. By addressing these multifaceted challenges, digital tools in rheumatology can play a pivotal role in bridging the digital divide and enhancing patient outcomes, ultimately contributing to the advancement of health equity in the field of rheumatology. Of note, none of the major chatbots is from a low-income group country and has limited or no functionality to support different demographics and nationalities.

As healthcare rapidly digitizes, incorporating technologies like mobile health apps, wearable devices, and AI, there is a pressing need for robust regulatory frameworks to prevent exacerbating health disparities (Table 5). This requires addressing deficiencies within existing regulations in regions like the EU and US to ensure the safe and effective use of DHT. Clear guidelines for health app approvals and wearable sensors are essential to unlock the full potential of digital medicine. Moreover, scrutinizing AI’s role in clinical decision-making is necessary to prevent algorithmic bias and ensure equitable healthcare delivery, especially for underserved populations. To effectively address these challenges, both LMICs and HICs need specific regulatory adjustments and healthcare delivery strategies to improve outcomes for RMD patients. Enhanced access to care, timely diagnosis, and consistent follow-up facilitated by DHT can significantly improve patient management if integrated effectively into the healthcare system.

**Figure 3:**
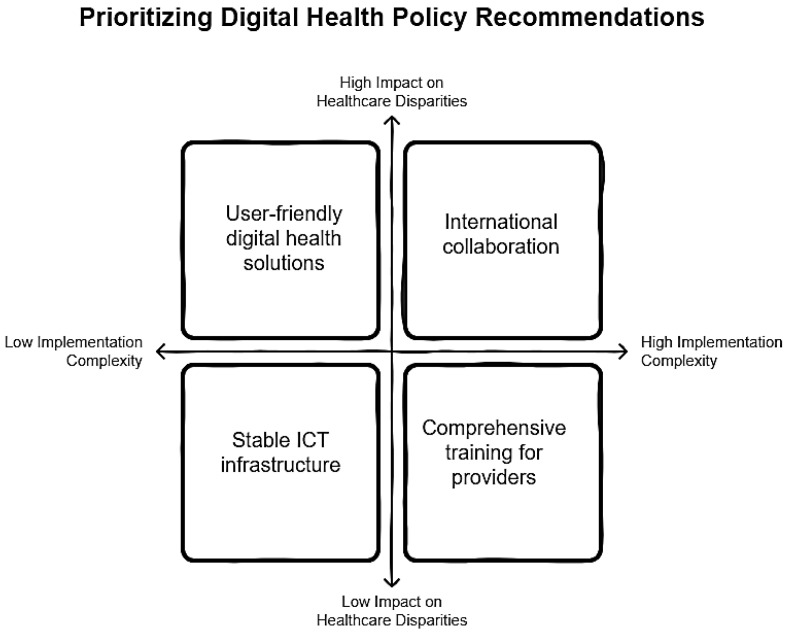
Digital Health Policy Recommendations. There are four focus areas: user-friendly solutions, international collaboration, stable infrastructure, and provider training, based on impact on disparities and implementation complexity.

To enhance the adoption of digital health solutions for managing RMDs across diverse healthcare settings, a comprehensive set of policy recommendations is necessary. Figure 3 illustrates the prioritization of Digital Health Policy Recommendations in a 2 × 2 matrix, suggesting four focus areas: user-friendly solutions, international collaboration, stable infrastructure, and provider training, based on impact on disparities and implementation complexity. These recommendations should address the unique challenges and opportunities in both LMICs and HICs. It is essential to ensure that digital health initiatives are developed with substantial input from end-users, including patients and healthcare providers, to guarantee that these tools address unmet needs and are tailored to enhance user engagement and adoption [29].

Figure 3 presents a schematic for setting priorities in digital health policies, focusing on two main factors: their impact on healthcare inequalities and how difficult they are to put into action. The section with high impact and low difficulty suggests starting with simple, easy-to-use digital health tools. On the other hand, the high-impact, high-difficulty section focuses on global partnerships, which require a lot of effort to coordinate but can greatly improve fairness in healthcare. The scheme also highlights the need for reliable technology systems, which are easier to set up but have a smaller direct effect on reducing inequalities. Additionally, training programs for healthcare providers, while costly and time-consuming, show limited immediate effects on reducing healthcare gaps. This approach helps policymakers decide where to focus resources to improve healthcare access and outcomes effectively. Overall recommendations can be summarized into the following points.

1. Focus on building a robust digital health ecosystem that supports stable electricity, ICT infrastructure, and affordable mobile internet services, especially in LMICs, to overcome infrastructural barriers [28].
2. Integrate digital health solutions into broader healthcare policies and ensure sustainable funding mechanisms, including the involvement of the private sector, to support long-term implementation and scalability [92].
3. Enhance governance structures to promote transparency, accountability, and public participation in digital health programs, utilizing ICTs to increase the effectiveness of these interventions [93].
4. Provide extensive training for healthcare providers and relevant stakeholders on the use of digital health tools and ensure that systems are interoperable to facilitate seamless data exchange and integration into clinical practice [94].
5. Ensure that digital health solutions are accessible and user-friendly for all population segments, particularly marginalized groups such as older adults and rural populations, to promote healthcare equity [95].
6. Use digital health applications like mNavigator that incorporate decision support algorithms and reminders to improve adherence to treatment protocols and management of diseases, as demonstrated in contexts like pediatric cancer care in Tanzania [96].
7. Foster international collaboration and learn from successful digital health implementations in both LMICs and HICs to drive innovation and improve digital health adoption globally [97,98].

By implementing these policy recommendations, governments and healthcare organizations can create an enabling environment that supports the effective adoption and scaling of digital health solutions. This will enhance the management of RMDs, improve patient outcomes, and contribute to the reduction of healthcare disparities across different regions.

## 5. Conclusions

The application of DHT in the management of rheumatic diseases holds immense potential to bridge the global disparities in rheumatic care. EHR, telemedicine, mobile health apps, and wearable devices offer promising tools to enhance disease monitoring, improve treatment adherence, and facilitate patient–provider communication. However, the successful integration of these technologies into rheumatic care faces significant challenges, particularly in LMICs compared to HICs. The digital divide, characterized by unequal access to technology, limited digital literacy, and infrastructure deficiencies, disproportionately affects LMICs and hinders the effective implementation of digital health solutions in these settings. To fully harness the potential of digital health in rheumatic care, a comprehensive policy framework is necessary. This framework should prioritize strategic investments in digital infrastructure, capacity building for healthcare professionals specializing in rheumatology, and the development of context-specific, user-centered digital health interventions tailored to the needs of patients with rheumatic conditions. Furthermore, fostering multi-stakeholder collaborations among governments, healthcare institutions, technology providers, and patient organizations is crucial to ensure the sustainable and equitable implementation of DHT in rheumatic care. By actively engaging diverse perspectives and experiences, we can develop a nuanced understanding of the unique challenges and opportunities in different settings, enabling the creation of targeted strategies that effectively address the needs of individuals with rheumatic diseases across the socioeconomic spectrum. Ultimately, by adopting a holistic, equity-focused approach to digital health integration in rheumatology, we can work towards a future where innovative technologies are leveraged to transform rheumatic care delivery, improve health outcomes, and alleviate the global burden of these chronic conditions. It is through concerted, collaborative efforts that we can build a more inclusive and resilient global health system, one that harnesses the power of digital health to ensure that no individual with rheumatic disease is left behind, regardless of their geographic location or socioeconomic status.

## Figures and Tables

**Table 1:** Overview of Digital Health Applications for Rheumatology: Key Features and Accessibility.

App Name	Key Features	Target User Group	Effectiveness Indicators
RheumaHelper	Diagnostic tools Disease activity calculators Medication guidelines	Healthcare professionals, patients	High user rating: 4.7 Strong adoption: 50 K+ installations
Manage My Pain	Pain tracking Report sharing with doctors	Patients managing pain	Good user rating: 4.1/4.7 (App Store) Highest adoption: 100 K+ installations
RA Monitor	Tracks RA symptoms Treatment responses Provider communication	Patients with rheumatoid arthritis	Good user rating: 4.4 Moderate adoption: 10 K+ installations
Arthritis Tracker	Symptom tracking Medication effects Personalized advice Report sharing	Patients with arthritis	Moderate adoption: 10 K+ installations
RheumaBuddy	Tracks RA symptoms Data sharing with healthcare professionals	Patients with rheumatoid arthritis	Moderate adoption: 10,000+ installations
My Arthritis	Symptom tracking Medication reminders Educational content	Patients with arthritis	Initial adoption: 5 K+ installations
ArthroCoach App	Exercise programs Educational content Progress tracking	Patients with arthritis	Initial adoption: 5 K+ installations
Care Arthritis Mobile	Educational content News dissemination for rheumatologists	Rheumatologists	Specialized use: 100+ installations

**Table 2:** Comparative Analysis of DHT in High-Income and Low- to Middle-Income Countries.

Implementation Aspects	HICs	LMICs
Infrastructure & Systems	EHRs (e.g., RISE registry) Telemedicine platforms Mobile health apps Wearable devices	Mobile health apps Basic telemedicine systems Limited EHR implementation Select wearable devices
Systemic Barriers	Integration challenges between digital tools User engagement issues Technological disparities among populations	Inadequate healthcare infrastructure High technology deployment costs Digital literacy barriers
Current Applications & Success Cases	RISE registry for quality measurement Adhera for real-time monitoring Semi-automated telemedicine referrals	ePROs implementation Rheumatic? digital diagnostic tools Rural telemedicine programs
Development Priorities	Integration of existing systems Enhanced user interface design Policy-driven technological equity	Infrastructure development Local language/cultural adaptation Digital literacy programs
Strategic Directions	Developer–clinician–patient collaboration EHR capability enhancement AI and big data integration	Technology access improvement International partnership utilization Resource sharing initiatives
Category	HICs	LMICs
Key Technologies	EHRs (e.g., RISE registry), telemedicine, mobile health apps, wearable devices	Mobile health apps, telemedicine, EHRs, wearable devices
Current Applications	EHRs enhance data quality and patient management; telemedicine reduces wait times and improves care delivery; wearable devices enable continuous health monitoring	Mobile apps for symptom tracking and disease management; telemedicine expands access to healthcare services
Challenges	Inadequate integration of digital tools; poor user engagement; technological disparities	High costs of technology deployment; inadequate healthcare infrastructure; digital literacy barriers
Opportunities for Improvement	Improve integration and user interface of digital tools; enhance patient engagement strategies; address technological disparities through policy and funding	Increase investment in healthcare infrastructure; adapt digital tools to local languages and cultures; implement educational programs to improve digital literacy
Potential Improvements	Semi-automated systems like telemedicine referrals to improve efficiency; use of AI and big data for personalized care	REMORA app integration for better clinical decision-making; real-time monitoring platforms for ongoing patient management
Success Examples	RISE registry for quality measurement; Adhera for Rheumatology for real-time disease outcome monitoring	Use of ePROs and digital diagnostic tools like Rheumatic? for early diagnosis; telemedicine programs reducing the need for travel in rural areas
Future Directions	Foster collaboration between developers, clinicians, and patients; further develop EHR capabilities and integration	Address the digital divide by ensuring access to necessary technologies; leverage international partnerships for resource sharing

**Table 3:** Comparative Analysis of Digital Health Implementation and Challenges in High-Income and Low- to Middle-Income Countries.

Aspect	HICs	LMICs
**Digital Health Technologies**	ePROs, telehealth, advanced EHRs	Basic telehealth, emerging ePROs, limited EHR capabilities
**Current Utilization**	Extensive integration of digital tools for real-time monitoring and remote consultations; successful management of disease activity and reduction of in-person visits	Emerging use of digital tools, often hindered by infrastructural challenges; less frequent and effective use of real-time data for disease management
**Key Challenges**	Ensuring the integration and user engagement with digital tools; addressing data security and privacy concerns	Shortage of rheumatologists and healthcare resources; lower levels of digital literacy and infrastructure deficits
**Opportunities for Improvement**	Enhance data security measures; improve patient and provider digital literacy and engagement	Increase support and training for digital tool usage; develop infrastructure to support digital health technologies
**Recommendations**	Develop standardized guidelines for digital health to ensure safe and effective use; foster patient-centered approaches and enhance data privacy protocols	Leverage international collaborations for resource and knowledge sharing; tailor digital solutions to local needs and capabilities
**Successful Initiatives**	Implementation of EULAR recommendations for telemedicine; use of digital registries like RABBIT SpA for innovative treatment strategies	Training programs by international organizations like EULAR to boost digital literacy; initiatives to integrate basic telehealth solutions in routine care
**Future Directions**	Continued advancement in AI and big data to personalize treatment and improve outcomes; expansion of remote patient monitoring systems	Development of locally adapted digital health tools; strengthening partnerships to enhance digital infrastructure

**Table 4:** Digital Health Tools for Rheumatology: A Global Overview of Applications and Features.

Tool Name	Type	Country	Rheumatology Disease	Key Features	Target User Group
ArthritisID	Mobile app	USA	Rheumatoid Arthritis	Symptom tracking, treatment information	Patients
Lupus Tracker	Mobile app	USA	Systemic Lupus Erythematosus	Flare tracking, medication reminders	Patients
MyLupusTeam	Social network	USA	Systemic Lupus Erythematosus	Community support, resources sharing	Patients
PsoriasisConnect	Social network	USA	Psoriatic Arthritis	Community forums, expert Q&A	Patients
Gout Central	Patient education web	USA	Gout	Disease education, dietary advice	Patients, educators
Track + React	Mobile app	USA	Various Rheumatic Diseases	Activity logging, reaction tracking	Patients
MySpA	Mobile app	UK	Ankylosing Spondylitis	Symptom tracking, treatment options	Patients
RheumInfo	Patient education web	Canada	Various Rheumatic Diseases	Treatment guidelines, drug information	Healthcare professionals
Rheuma Auszeit	Mobile app	Germany	Various Rheumatic Diseases	Relaxation techniques, disease management	Patients
Ouchie	Mobile app	USA	Juvenile Idiopathic Arthritis	Pain management, reward system	Young patients
Sjögren’s Tracker	Mobile app	USA	Sjögren’s Syndrome	Symptom journaling, hydration tracking	Patients
RheumaBuddy	Mobile app	Denmark	Various Rheumatic Diseases	Community interaction, daily logging	Patients
Psoriasis Association	Patient education web	UK	Psoriatic Arthritis	Latest research, treatment options	Patients, researchers
SingHealth RheumConnect	Rule-based chatbot	Singapore	Autoimmune Rheumatic Diseases (AIRD)	The chatbot is available 24/7 at no cost	Patients

**Table 5:** Global Healthcare Challenges and Technological Solutions.

Aspect	Challenges in LMICs	Challenges in HICs	Technological Advancements	Regulatory Needs
Healthcare Infrastructure	Insufficient healthcare infrastructure, delayed diagnoses, lack of specialists	Better resources but need for improved guideline implementation	EHRs, biobanking, AI-based diagnostics improve diagnosis and treatment	Robust frameworks to ensure safe, effective technology use
Clinical Guidelines	Implementation issues, integration of lifestyle management	Same as LMICs but with resources for better implementation	Digital tools including REMORA app to support guideline adherence	Guidelines for health apps, wearables, and AI use
Opioid Management	Varying definitions and recognition of long-term use	Concerns over opioid dependence and policy consistency		Clear standards for long-term opioid use in RMD care
Patient-Centered Care	Scarcity of specialized caregivers	Well-equipped but needs better patient engagement strategies	Role of AI and mobile apps (e.g., REMORA) in enhancing engagement	Regulations to prevent algorithmic bias, ensure equity
Collaborative Efforts	Need for global best practice exchange	Same as LMICs, with resources to lead collaborations	Platforms for global collaboration and data sharing	Support for international partnerships and policy alignment

## Data Availability

The article is a perspective piece and does not include any original data. All information discussed in this article is based on previously published studies and publicly available sources, which have been cited throughout the text.

## Abbreviations

AI	Artificial intelligence
BAME	Black, Asian, and Minority Ethnic
COmPASS	Capturing Outcomes for People with Ankylosing Spondylitis Study
DHT	Digital health technologies
EHR	Electronic health records
EULAR	European Alliance of Associations for Rheumatology
HIC	High-income countries
ICT	Information and communication technology
IIM	Idiopathic inflammatory myopathies
IoMT	Internet of Medical Things
IT	Information technology
LMIC	Low- and middle-income countries
ML	Machine learning
NGO	Non-governmental organizations
RA	Rheumatoid arthritis
RABBIT	Rheumatoid Arthritis Observation of Biologic Therapy
REMORA	Rheumatology Database and Biobank
RHD	Rheumatic heart disease
RISE	Rheumatology Informatics System for Effectiveness
RMD	Rheumatic and musculoskeletal disease
SCQM	Swiss Clinical Quality Management in Rheumatic Diseases
SLE	Systemic lupus erythematosus
TAK	Takayasu arteritis
TELERA	Telemonitoring of Rheumatoid Arthritis
TNF	Tumor necrosis factor
WebRA	Web-based Rheumatoid Arthritis
